# Phylogenetic analysis of mitochondrial substitution rate variation in the angiosperm tribe *Sileneae*

**DOI:** 10.1186/1471-2148-9-260

**Published:** 2009-10-31

**Authors:** Daniel B Sloan, Bengt Oxelman, Anja Rautenberg, Douglas R Taylor

**Affiliations:** 1 Department of Biology, University of Virginia, Charlottesville, VA, USA; 2 Department of Plant and Environmental Sciences, University of Gothenburg, Gothenburg, Sweden; 3 Department of Systematic Biology, EBC, Uppsala University, Uppsala, Sweden

## Abstract

**Background:**

Recent phylogenetic studies have revealed that the mitochondrial genome of the angiosperm *Silene noctiflora *(Caryophyllaceae) has experienced a massive mutation-driven acceleration in substitution rate, placing it among the fastest evolving eukaryotic genomes ever identified. To date, it appears that other species within *Silene *have maintained more typical substitution rates, suggesting that the acceleration in *S. noctiflora *is a recent and isolated evolutionary event. This assessment, however, is based on a very limited sampling of taxa within this diverse genus.

**Results:**

We analyzed the substitution rates in 4 mitochondrial genes (*atp1*, *atp9*, *cox3 *and *nad9*) across a broad sample of 74 species within *Silene *and related genera in the tribe *Sileneae*. We found that *S. noctiflora *shares its history of elevated mitochondrial substitution rate with the closely related species *S. turkestanica*. Another section of the genus (*Conoimorpha*) has experienced an acceleration of comparable magnitude. The phylogenetic data remain ambiguous as to whether the accelerations in these two clades represent independent evolutionary events or a single ancestral change. Rate variation among genes was equally dramatic. Most of the genus exhibited elevated rates for *atp9 *such that the average tree-wide substitution rate for this gene approached the values for the fastest evolving branches in the other three genes. In addition, some species exhibited major accelerations in *atp1 *and/or *cox3 *with no correlated change in other genes. Rates of non-synonymous substitution did not increase proportionally with synonymous rates but instead remained low and relatively invariant.

**Conclusion:**

The patterns of phylogenetic divergence within *Sileneae *suggest enormous variability in plant mitochondrial mutation rates and reveal a complex interaction of gene and species effects. The variation in rates across genomic and phylogenetic scales raises questions about the mechanisms responsible for the evolution of mutation rates in plant mitochondrial genomes.

## Background

**Figure 1:**
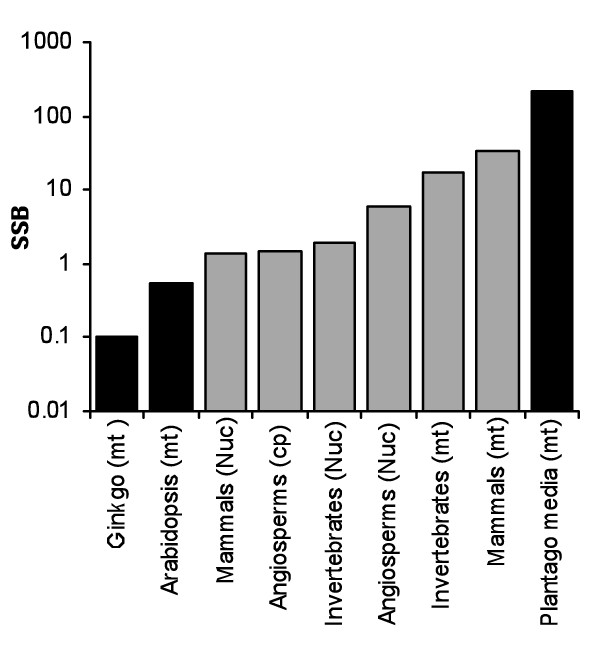
**Diversity in substitution rates**. Synonymous substitution rates per site per billion years (SSB) for different organisms and genomes plotted on a log scale. Black bars represent seed plant mitochondrial genomes. Average rates for animal taxa from Lynch *et al*. [20]; angiosperm chloroplast and nuclear estimates from Wolfe *et al*. [74]; mitochondrial rates for individual plant species taken from Cho *et al*. [5] and Mower *et al*. [4].

Substitution rates in plant mitochondrial genomes are generally low relative to their nuclear and chloroplast counterparts, as well as relative to the mitochondrial genomes of other organisms [1-3]. In fact, absolute rates of sequence evolution in seed plant mitochondrial DNA (mtDNA) are among the slowest ever estimated (Figure 1; [4]). A series of recent studies, however, has revealed notable exceptions to this generalization [4-7]. There are angiosperm species that not only deviate from the slow substitution rates typical of plant mtDNA but also exhibit some of the highest eukaryotic substitution rates ever documented (Figure 1). With such a substantial fraction of known rate variation captured in a relatively small twig within the tree of life, plant mitochondrial genomes represent an intriguing system for investigating the evolutionary forces that shape substitution rates [8-14].

Studies of rate accelerations in plant mitochondrial genomes have consistently shown that these effects are most pronounced at so-called synonymous sites, which do not affect the corresponding amino acid sequence (*e.g*. [5]). One of the pillars of the neutral theory of molecular evolution is that the rate of neutral substitutions (*i.e*. those with no fitness effect) is expected to equal the mutation rate [15]. Synonymous substitutions are not completely neutral, however. They are subject to a variety of selection pressures including translational efficiency, mRNA stability and the conservation of regulatory motifs (reviewed in [16]), and direct measurements of mutation rates can be more than an order of magnitude higher than those estimated from synonymous substitution rates [17]. Nevertheless, synonymous sites still offer one of our best approximations of the underlying mutation rate. Therefore, considering the absence of well-supported alternative hypotheses, the extreme synonymous substitution rates observed in certain plant mitochondrial genomes are most likely a result of mutational acceleration.

*Silene noctiflora *(Caryophyllaceae) is a recent addition to a growing list of angiosperms exhibiting major accelerations in mitochondrial synonymous substitution rate [4,7]. In other well-documented examples (*e.g. Plantago *and *Pelargonium*), rate accelerations appear relatively old (*ca*. 30-80 million years) having preceded the divergence of large clades or even an entire genus [6]. In contrast, the extreme mitochondrial substitution rates of *S. noctiflora *appear unique relative to other *Silene *species, suggesting a very recent acceleration. Estimates of mitochondrial substitution rate, however, are available for only a few *Silene *species, representing a tiny fraction of this large and diverse genus. The sparse sampling severely limits the phylogenetic resolution to detect historical changes in substitution rate.

The scarcity of mitochondrial sequence data within *Silene *reflects a broader under-representation of plant mtDNA in studies of molecular evolution. Whereas chloroplast DNA (cpDNA) and animal mtDNA are utilized extensively in phylogenetic studies, the low baseline substitution rates and growing evidence for rate heterogeneity in plant mtDNA often limit its utility in this context--particularly at local phylogenetic scales [18]. Understanding the causes and consequences of mutation rate variation is a fundamental problem in evolutionary biology [12,19-21], but the lack of plant mtDNA sequence data is a hindrance to investigating this question. To characterize the pattern of mitochondrial substitution rate variation throughout *Silene *and related genera, we sequenced four mitochondrial loci in a sample of 74 species that were selected to capture the phylogenetic diversity of this genus and its closest relatives (Table 1). To our knowledge, this effort represents the most extensive species-level sampling to date of mitochondrial sequence divergence in a plant genus.

**Table 1:** Sampled species and voucher information.

**Species**	**Voucher**
*Agrostemma githago *L.	D. Sloan 001 (VPI)
*Atocion lerchenfeldianum *(Baumg.) M. Popp	Strid 24875 (GB)
*Eudianthe laeta *(Aiton) Rchb. ex Wilk.	Strandhede *et al*. 690 (GB)
*Heliosperma pusillum *(Waldst. & Kit.) Rchb.	E. Zogg ZH 1438 (Z)
*Lychnis coronaria *(L.) Desr.	N/A. Collected by D. Sloan. Charlottesville, VA, USA
*Petrocoptis pyrenaica *A.Br.	Schneeweiss *et al*. 6549 (WU)
*Silene acaulis *(L.) Jacq.	*Schneeweiss 5315 (WU)
*Silene acutifolia *Link ex Rohrb.	Rothmaler 13691 (S)
*Silene akinfievii *Schmalh.	Portenier 3814 (LE)
*Silene ammophila *Boiss. & Heldr.	Raus 7631 (GB)
*Silene antirrhina *L.	N/A. Collected by D. Sloan. Kellog, MN, USA
*Silene argentina *(Pax) Bocquet	M. Popp 2005-11-11 (GB)
*Silene armena *Boiss.	B. Oxelman 2436 (GB)
*Silene auriculata *Sibth. & Sm.	Baden & Franzén 795 (Strid)
*Silene bellidifolia *Jacq.	Strid *et al*. 35179 (Strid)
*Silene caesia *Sm.	Baden 1114 (Strid)
*Silene caryophylloides *(Poir) Otth	Görk *et al*. 2436 (Strid)
*Silene ciliata *Pourr.	Franzén *et al*. 822 (Strid)
*Silene conica *L.	P. Erixon 70 (UPS)
*Silene conoidea *L.	A. Rautenberg 290 (GB)
*Silene cordifolia *All.	Lippert & Merxmüller 17265 (Strid)
*Silene davidii *(Franch.) Oxelman & Lidén	F. Eggens 85 (UPS)
*Silene delicatula *Boiss.	B. Oxelman 2456 (GB)
*Silene dichotoma *Ehrh.	W. Till 17.7.2004 (WU)
*Silene douglasii *var. *oraria *(M. Peck) C.L. Hitchc. & Maguire	*N/A. Collected by S. Kephart. Cascade Head, OR, USA
*Silene flavescens *Waldst. & Kit.	Strid & Papanicolaou 15820 (Strid)
*Silene fruticosa *L.	B. Oxelman & Tollsten 934 (GB)
*Silene gallica *L.	D. Sloan 002 (VPI)
*Silene gallinyi *Heuff. ex Rchb.	Strid & Hansen 9283 (Strid)
*Silene gracilicaulis *C.L. Tang	Smith 11346 (UPS)
*Silene hookeri *Nutt. subsp. *hookeri*	F. Schwartz 107 (WTU)
*Silene imbricata *Desf.	B. Oxelman 1881 (GB)
*Silene integripetala *Bory & Chaub.	B. Oxelman 1902 (GB)
*Silene involucrata *(Cham. & Schltdl.) Bocquet	F. Eggens 7 (UPS)
*Silene khasyana *Rohrb.	Einarsson et.al 3025 (UPS)
*Silene lacera *(Stev.) Sims	Schönswetter & Tribsch Iter Georgicum 51 (WU)
*Silene laciniata *subsp. *californica *(Durand) J.K. Morton	Schwartz 102-2 (WTU)
*Silene latifolia *Poir.	*N/A. Collected by J. Greimler. Vienna, Austria
*Silene littorea *Brot.	P. Erixon 74 (UPS)
*Silene macrodonta *Boiss.	B. Oxelman 2441 (GB)
*Silene menziesii *Hook.	Kruckeberg 3436 (WTU)
*Silene moorcroftiana *Wall. ex Benth	B. Dickoré 17783 (Dickoré)
*Silene multicaulis *Guss.	Strid & Hansen 9954 (Strid)
*Silene muscipula *subsp. deserticola Murb.	*Chevalier 548 (WU)
*Silene nana *Kar. & Kir.	Kereverzova & Mekeda 1976.V.5 (LECB)
*Silene nicaeensis *All.	D. Sloan 005 (VPI)
*Silene noctiflora *L.	D. Sloan 003 (VPI)
*Silene nutans *L.	*Larsen, Larsen & Jeppesen 196 (S)
*Silene odontopetala *Fenzl	Görk *et al*. 23817 (Strid)
*Silene otites *(L.) Wibel	A. Rautenberg 83 (UPS)
*Silene paradoxa *L.	W. & S. Till 21 July 2002 (WU)
*Silene paucifolia *Ledeb.	H. Solstad & Elven 04/1384 (O)
*Silene pendula *L.	A. Rautenberg 289 (GB)
*Silene pygmaea *Adams	Amirkhanov 22.VI-1977 MW)
*Silene repens *Patrin	Argus 1068 (UPS)
*Silene sachalinensis *F. Schmidt	Popov 1949.VII.8 (LE)
*Silene samia *Melzh. & Christod	B. Oxelman 2208 (UPS)
*Silene samojedora *(Sambuk) Oxelman	H. Solstad, R. Elven SUP-04-3871 (O)
*Silene schafta *S.G. Gmel. ex Hohen.	M. Popp 1053 (UPS)
*Silene schwarzenbergeri *Halácsy	Hartvig & Christiansen 8167 (Strid)
*Silene seoulensis *Nakai	Hong & Han 13420001 (UPS)
*Silene sordida *Hub.-Mor. & Reese	B. Oxelman 2206 (GB)
*Silene sorensenis *(B. Boivin) Bocquet	F. Eggens 48 (UPS)
*Silene stellata *(L.) W.T. Aiton	N/A. Collected by D. Sloan. Giles County, VA, USA
*Silene succulenta *Forssk.	Strid & Kit Tan 55028 (Strid)
*Silene tunicoides *Boiss.	Carlström 5970 (Strid)
*Silene turkestanica *Regel	K. Kiseleva 20.VI.1970 (MW)
*Silene uniflora *Roth	P. Erixon 73 (UPS)
*Silene vittata *Stapf	B. Oxelman 2390 (UPS)
*Silene vulgaris *(Moench) Garcke	*N/A. Collected by M. Dzhus. Minsk, Belarus
*Silene yemensis *Deflers	Hepper 5792 (WU)
*Silene zawadzkii *Herbich	B. Oxelman 2241 (GB)
*Viscaria alpina *(L.) G. Don	B. Frajman & Schönswetter 11415 (LJU)
*Viscaria vulgaris *Bernh.	P. Schönswetter & B. Frajman 11097 (LJU)

Herbaria abbreviations are from Holmgren *et al*. [75], except Dickoré (private herbarium Bernhard Dickoré, Göttingen, Germany), and Strid (private herbarium Arne Strid, Ørbaek, Denmark). Asterisks indicate that a second specimen was used for one or more loci [see Additional file 9].

To compare absolute substitution rates in a gene across lineages requires an estimate of the genealogy with dated nodes (*i.e*. divergence times). In cases of extreme rate variation, generating such a tree directly from the gene in question is problematic. With rate variation, slowly-evolving taxa can be difficult to resolve, and long branch attraction can favor incorrect topologies [22]. Even with an accurate topology, rate variation can bias the estimate of divergence times with molecular clock based methods. For this reason, previous studies of substitution rate variation in plant mitochondrial genomes have constrained their analyses based on phylogenies and divergence times inferred from nuclear and chloroplasts sequences.

Because both mitochondrial and chloroplast genomes are predominantly maternally inherited in *Silene*, they are expected to share a common genealogy [23,24] (although breakdowns in uniparental inheritance may potentially disrupt this relationship [25-27]). Therefore, we chose the chloroplast gene *matK *to estimate phylogenetic relationships and divergence times. This gene has proven to be highly informative in phylogenetic reconstruction, partly because of its high rates of substitution [28,29]. It has also been used in two recent analyses of divergence times within *Silene *and the Caryophyllaceae [4,30].

We identified substantial rate accelerations in multiple lineages within the *Silene *phylogeny as well as major rate differences among mitochondrial genes. Here, we discuss the complex patterns of mitochondrial rate variation in the genus *Silene *and the implications they have for the evolution of mitochondrial mutation rates and the patterns of selection on mtDNA at the sequence level.

## Results

### Chloroplast DNA phylogeny

**Figure 2:**
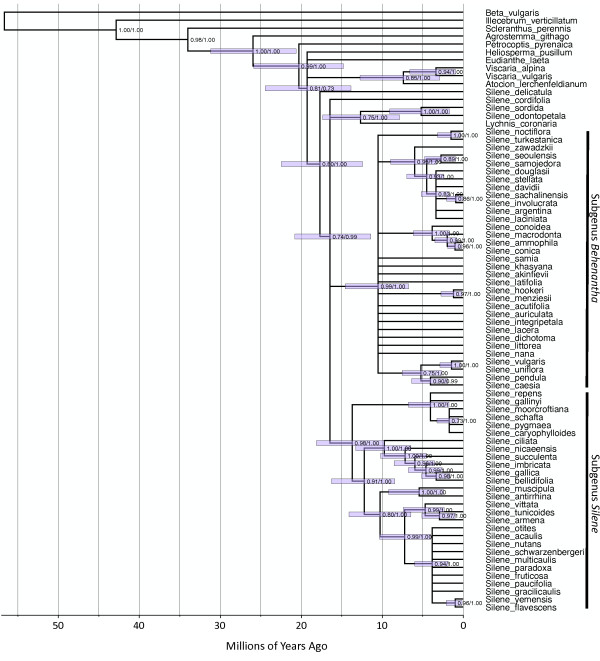
**Chronogram showing divergence times estimated in BEAST based on full-length *matK *coding sequence**. Time scale is in millions of years. Error bars at each node show 95% HPD for node age. Values to the right of each node show Bayesian posterior probability and parsimony bootstrap support (in that order) for the corresponding clade. Tree topology was constrained based on 70% parsimony bootstrap consensus.

Likelihood, parsimony and Bayesian phylogenetic methods were in general agreement for the *matK *dataset. The 70% parsimony bootstrap consensus tree (Figure 2) did not conflict with any of the nodes from either the ML or Bayesian analysis. The results were also generally consistent with previous cpDNA studies of the tribe *Sileneae *[31,32]. The analysis recovered the two previously identified subgenera (*e.g*. [32])--*Silene *and *Behenantha *(Otth.) Endl. (=subgenus *Behen *(Dumort.) Rohrb.)--along with the relationships among the major clades in subgenus *Silene*. There was, however, incomplete resolution in some parts of the tree--particularly among the major lineages within subgenus *Behenantha*, which appear as a large radiation. Four *Silene *species were not grouped with either of the two major subgenera. As found in the analysis of other chloroplast loci, *S. sordida *was placed in a clade with *Lychnis *[32]. *Silene odontopetala *was also assigned to this clade with strong support. The relationships between subgenus *Behenantha*, subgenus *Silene*, the *Lychnis*/*S. odontopetala*/*S. sordida *clade, and a fourth lineage consisting solely of *S. cordifolia *could not be confidently resolved. Finally, there was unexpected support for a sister relationship between *S. delicatula *and the rest of our *Silene*/*Lychnis *sample.

### Divergence times

We used three different dating methods, which produced roughly similar estimates of divergence times, but there was a consistent pattern distinguishing them [see Additional files 1 and 2]. Specifically, the Langley-Fitch method produced the youngest estimates of divergence times within *Sileneae*, while a penalized likelihood method produced the oldest. For example, the estimated age of the root node for the entire *Silene*/*Lychnis *clade differed by 50% between the two methods (21.0 vs. 14.0 Myr). The BEAST model (Figure 2) generally produced intermediate estimates of divergence time relative to the other two methods. Only the BEAST values were used for subsequent rate analyses, so the uncertainty in divergence times should be considered when interpreting absolute substitution rate estimates.

### Mitochondrial rate variation

**Figure 3:**
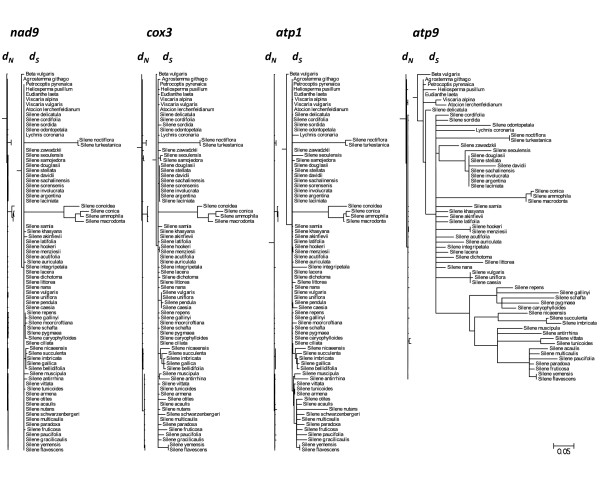
***d*_*N *_and *d*_*S *_trees for mitochondrial genes**. Branch lengths are in terms of non-synonymous (*d*_*N*_) or synonymous (*d*_*S*_) substitutions per site as estimated by PAML under a constrained topology. The scale is the same for all trees.

Branch lengths in terms of both synonymous (*d*_*S*_) and non-synonymous (*d*_*N*_) substitutions per site for each mitochondrial gene are shown in Figure 3. All four genes show little divergence at non-synonymous sites across the entire tree (Table 2). In addition, they all share a pattern of extreme synonymous divergence in six *Silene *species that can be divided into two clear clades: (1) the previously characterized *S. noctiflora *along with its close relative *S. turkestanica *and (2) *S. ammophila*, *S. conica*, *S. conoidea*, and *S. macrodonta*, which all belong to section *Conoimorpha*. Beyond those similarities, the four mitochondrial genes differ markedly in synonymous branch lengths (Figure 3). Very little divergence is observed in *nad9 *outside of the aforementioned six species. Synonymous divergence is similarly low throughout much of the *cox3 *and *atp1 *trees, but there are a number of species that exhibit substantial divergence, particularly within subgenus *Silene*. This group includes *S. nutans *which, despite showing no sign of abnormal divergence in *cox3 *and *nad9*, is highly divergent for *atp1*. Finally, synonymous divergence in *atp9 *is extreme and highly variable throughout most of the genus *Silene*, although many of the outgroup genera exhibit typically low levels of divergence. The total synonymous tree length is approximately 9-fold larger for *atp9 *than the slowly-evolving *nad9*. This gap widens to 41-fold if the six taxa that have accelerated rates across all genes are excluded from the analysis (Table 2).

**Table 2:** Absolute substitution rates by gene (SSB).

	***R*_*N*_**	***R*_*S*_**	***ω***
	
*nad9 *(378 bp)	0.36 (0.18)	2.62 (0.51)	0.137 (0.357)
*cox3 *(588 bp)	0.38 (0.22)	3.43 (1.64)	0.110 (0.133)
*atp1 *(960 bp)	0.20 (0.13)	4.25 (2.38)	0.048 (0.055)
*atp9 *(162 bp)	0.39 (0.41)	22.66 (20.75)	0.017 (0.020)

Values represent tree-wide average rates (total branch length divided by total branch time) based on the subset of 61 species for which we have sequence for all 4 mitochondrial genes. The values in parentheses are the rates estimated after excluding the two clades with highly elevated rates across all 4 genes.

**Figure 4:**
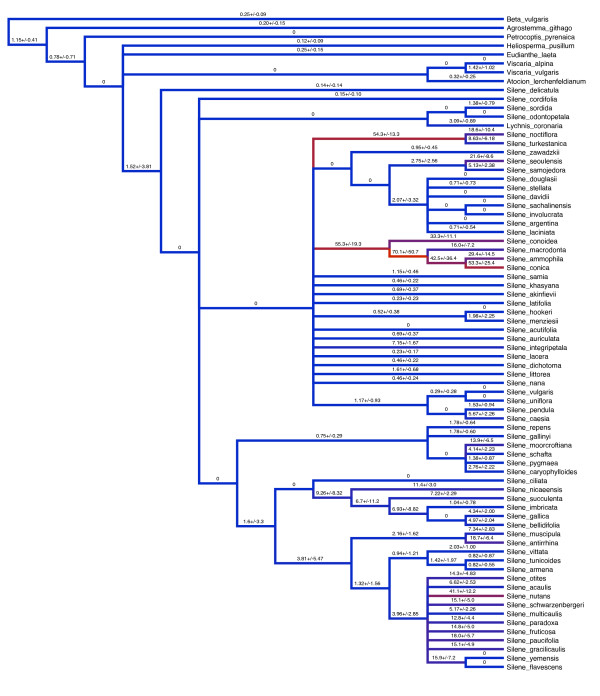
**Phylogenetic variation in *R*_*S*_**. Branches labelled with absolute synonymous substitution rates and approximate standard errors based on concatenation of *nad9*, *cox3 *and *atp1*. Branch colors indicate fast (red) and slow (blue) rates.

As expected given the enormous variation in mitochondrial divergence across species, absolute synonymous substitution rates (*R*_*S*_) differ dramatically throughout the tribe *Sileneae *(Figure 4, [see Additional files 3 and 4]). The outgroups to *Silene *tend to have *R*_*S *_values of less than 0.5 substitutions per site per billion years (SSB), and certain branches have an estimated *R*_*S *_of 0 because they lack a single synonymous substitution (Figure 4). Many lineages within *Silene *have maintained these low rates. At the other extreme, the rapidly-evolving *Silene *lineages have *R*_*S *_values that are more than two orders of magnitude greater than the low rates of *Beta vulgaris *and other outgroups. The fastest rate estimates observed in the entire dataset were found in the *atp9 *tree. The internal branch subtending the minimally inclusive clade that contains *S. succulenta *and *S. imbricata *had an estimated *R*_*S *_value of 392 SSB. The fastest terminal branch in the *atp9 *tree was that of *S. schafta *with a rate of 292 SSB, although it should be noted that the error associated with *atp9 *rate estimates for individual branches was generally large [see Additional file 4]. *R*_*S *_and *R*_*N *_values were both positively correlated across *atp1*, *cox3 *and *nad9*, but this correlation broke down in comparisons with *atp9 *and *matK *(Table 3). In addition, *R*_*S *_and *R*_*N *_values were significantly correlated with each other within genes for *atp1*, *cox3 *and *nad9 *but not for the other two loci.

**Table 3:** Pairwise *R*_*N *_and *R*_*S *_correlation coefficients within and among genes across phylogenetic lineages

	***nad9***	***cox3***	***atp1***	***atp9***	***matK***
	
***nad9***	**0.86**	**0.88**	**0.73**	0.04	0.17
***cox3***	**0.52**	**0.49**	**0.77**	0.11	0.15
***atp1***	**0.39**	**0.65**	**0.70**	0.13	0.13
***atp9***	-0.11	-0.12	-0.12	0.12	-0.05
***matK***	0.03	-0.03	-0.02	0.13	0.28

Values above and below diagonal are from pairwise comparisons between genes for *R*_*S *_and *R*_*N*_, respectively. Values on the diagonal are correlation coefficients between *R*_*N *_and *R*_*S *_within each gene. Bold values are significantly different from 0 based on a Bonferroni corrected *α *of 0.05/25 = 0.002.

### Evolutionary congruence between mitochondrial and chloroplast genomes

We utilized a constrained topology derived from cpDNA to analyze evolutionary rates in mtDNA, reflecting the assumption that the two organelle genomes share a single genealogy. Although the mitochondrial genes often yielded limited phylogenetic signal because of the dual problems of low variation and long branch attraction, there was some evidence to support phylogenetic congruence between these genomes [see Additional file 5]. For example, *S. hookeri *and *S. menziesii *were consistently paired by both chloroplast and mitochondrial genes, suggesting that the allopolyploid *S. hookeri *inherited both of its cytoplasmic genomes from the *S. menziesii *parental lineage [33]. In addition, the rapidly evolving *atp9 *gene produced a tree that generally agreed with the chloroplast *matK *topology at younger nodes, which are presumably less susceptible to saturation at synonymous sites.

There were a large number of incongruencies between chloroplast and mitochondrial trees, but they were generally lacking in support. Perhaps the most suspicious example of conflict between mitochondrial and chloroplast topologies was the placement of *S. samojedora*, *S. seoulensis*, *S. zawadzkii *and the major accelerated species from subgenus *Behenantha *in a clade otherwise populated by subgenus *Silene *in the *cox3 *tree [see Additional file 5]. Although this clade was supported by 74% of bootstrap replicates, inspection of the alignments showed that the grouping was based entirely on a single 6 bp region with 5 substitutions, raising doubts about the independence of those characters. Overall, we found no overwhelming evidence of conflicts between mitochondrial and chloroplast topologies, but the lack of mitochondrial divergence in many lineages gave us little statistical power. Therefore, it is possible that topological inaccuracies in our constraint tree could have led to misidentification of small mitochondrial rate accelerations, but given the phylogenetic scale of our analysis, it is unlikely that any of the major rate changes was an artifact of topological conflicts.

To date, studies of angiosperms with major increases in plant mitochondrial substitution rates (*e.g. Plantago*, *Pelargonium *and *Silene*) have concluded that the observed accelerations are largely independent of evolutionary rates in the chloroplast or nuclear genomes (although there is growing evidence for accelerated sequence and structural evolution in the chloroplast genomes of species with high mitochondrial substitution rates [34-36]). In our dataset, we found little substitution rate variation among species for the chloroplast *matK *gene and no significant correlation between mitochondrial and chloroplast rates (Table 3).

## Discussion

### Mutation rate variation among species

*Silene noctiflora *has been shown to have dramatically accelerated rates of mitochondrial evolution relative to its congeners [4,7]. We examined the phylogenetic distribution of this rate acceleration within the tribe *Sileneae *and identified six *Silene *species grouped into two clades that exhibited major increases in synonymous substitution rates across all four loci examined (Figure 3). As an illustration of the magnitude of these accelerations, we note the average synonymous pairwise divergence between these two closely-related clades within *Silene *subgenus *Behenantha *exceeds the divergence typically observed between flowering plants and liverworts--the deepest split in the land plant phylogeny [37]. Based on the currently available data in seed plants, the synonymous substitution rates exhibited by these rapidly-evolving lineages (Figure 4) are exceeded only by the fastest lineages of *Plantago *(Figure 1) [5]. In addition, the observed rates are on par with average estimates for mammalian mtDNA, although they still fall well below the fastest mammalian rates [38]. As discussed above (see Background), the observed differences in synonymous substitution rates most likely reflect differences in the underlying mutation rate.

The phylogenetic data remain ambiguous with respect to whether the two clades with rate accelerations represent independent evolutionary events. The *matK *tree does not strongly support or reject a monophyletic relationship between *S. noctiflora*/*S. turkestanica *and section *Conoimorpha *(Figure 2; [see Additional files 6 and 7]). More thorough phylogenetic analyses of these taxa have recently been conducted, utilizing both chloroplast and nuclear loci [39]. These studies have found that, while cpDNA sequences suggest phylogenetic independence between the two clades, at least some nuclear loci support monophyly. Therefore it is possible but inconclusive that both high rate clades are sister taxa that inherited an accelerated mitochondrial substitution rate from a common ancestor. If so, the two clades must have split shortly after that acceleration, because internal branches shared by the two lineages in the mitochondrial gene trees are quite short relative to the divergence between the lineages [see Additional file 5]. Resolving these phylogenetic relationships could prove difficult because previous studies have shown that the evolutionary history of subgenus *Behenantha *may be complicated by reticulation [32,40], such that relationships differ across genes and genomes

Comparisons of mitochondrial sequences from multiple populations of *S. noctiflora *have revealed very low levels of polymorphism, suggesting that the historically high mutation rates in this lineage may have undergone a reversion to more typical levels ([41] and unpublished data). This conclusion was, at least partially, supported by our phylogenetic data. The terminal branches for *S. noctiflora *and *S. turkestanica *exhibited a marked reduction in *R*_*S *_values relative to the ancestral rate for that clade (Figure 4). In contrast, the patterns of divergence within section *Conoimorpha *gave little indication of rate reversions.

The genus *Silene *is characterized by great diversity in breeding system and life history, and there has been substantial interest in how these traits may be related to molecular evolution in mitochondrial genomes [14,26,41-44]. There is no clear correlation between breeding system/life history and rate acceleration. The species exhibiting rate acceleration across all four mitochondrial genes are all hermaphroditic/gynomonoecious annuals with the exception of S. turkestanica, which is perennial. However, there are at least ten additional annual lineages represented in our sampling, and breeding system (hermaphroditic/gynomonoecious or gynodioecious) has yet to be determined for most species.

### Mutation rate variation among genes

Substitution rates commonly differ among regions within a genome because of variation in selection and/or mutational pressure, and a previous study had already identified substantial rate heterogeneity among *Silene *mitochondrial genes [45]. Nevertheless, the differences in synonymous substitution rates among mitochondrial genes in the current study are surprisingly large. If the six species that show universal acceleration across all four mitochondrial genes are excluded, *atp9 *appears to be evolving more than 40 times faster than *nad9 *at synonymous sites, while *cox3 *and *atp1 *fall in between these extremes.

The extreme elevation in *atp9 *substitution rates calls into question whether a biological mechanism other than an increase in the mutation rate might be responsible. The obvious alternatives to explain high levels of divergence include horizontal gene transfer (HGT) from distantly related species [46], maintenance of ancient, trans-specific polymorphism by balancing selection [26,41,44,47], re-localization of the gene to the higher mutation rate environment of the nuclear genome [48], or relaxed selection in a non-functional pseudogene [49].

None of these explanations, however, are fully consistent with the data. To explain the observed levels of divergence based on HGT without an increase in evolutionary rates would require multiple phylogenetically distant donor species (*i.e*. outside the angiosperms). Phylogenetic analysis of *atp9*, however, clearly places these sequences within the Caryophyllaceae ([Additional file 5] and unpublished data; note that this argument also applies to the lineage-specific divergence in *S. noctiflora*/*S. turkestanica *and section *Conoimorpha*). Likewise, in the absence of rate acceleration, an explanation based on balancing selection alone would require that polymorphism be maintained for hundreds of millions of years. Such a model seems extremely unlikely and even still could not explain the retention of partial phylogenetic congruence between *atp9 *and *matK*. Of course, the fact that balancing selection alone cannot explain the pattern of divergence in *atp9 *does not rule out the possibility that balancing selection has been acting on *atp9 *and other mitochondrial genes in *Silene*.

It is unlikely that *atp9 *has been functionally transferred to the nucleus in at least four *Silene *species--*S. latifolia*, *S. noctiflora*, *S. vulgaris *and *S. paradoxa*. Whole mitochondrial genome sequences confirm that *atp9 *is mitochondrially encoded in both *S. latifolia *and *S. noctiflora *(Sloan *et al*., unpublished data). In addition, the gene has been shown to be maternally inherited in *S. vulgaris *[26]. Comparing cDNA and genomic sequence also confirms that *atp9 *contains a site that undergoes C-to-U RNA editing in *S. paradoxa*--a process that is characteristic of organellar but not nuclear genes in plants (Sloan *et al*., unpublished data). Although we cannot definitively rule out the possibility of nuclear transfer, these data strongly suggest that nuclear transfer is not the driving force behind the pattern of elevated substitution rate observed in *atp9*.

It is also clear that *atp9 *is functional based on its low *ω *values and the absence of internal stop codons. Therefore, we conclude that the most likely explanation for the high levels of divergence is an increased mutation rate that is specific to *atp9 *(or a subset of the mitochondrial genome that includes *atp9*).

The molecular evolution of *atp9 *could be influenced by the presence of multiple gene copies in at least some species (see Methods). The existence of multiple copies could reflect heteroplasmy resulting from paternal leakage [25], non-functional paralogs in the mitochondria or other genomes [50], or the existence of multiple functional mitochondrial copies [51]. It is conceivable that *atp9 *is located in a region of active recombination within the *Silene *mitochondrial genome or is experiencing frequent retroprocessing back into the genome from mRNA. Both of these processes may be mutagenic as well as lead to gene duplication and, therefore, would be consistent with our observations [6,52,53]. Alternatively, high mutations rates in *atp9 *may have simply increased divergence between heteroplasmic and/or paralogous copies, thereby enhancing our ability to detect multiple copies of *atp9 *even though they exist for other genes as well. Sequencing complete mitochondrial genomes, analyzing relative copy number of *atp9 *variants, and sampling multiple individuals per species would help distinguish between these possibilities. In a sample of individuals from 40 different populations of *S. vulgaris*, we found 4 individuals with multiple *atp9 *copies, and certain variants were only found in multi-copy individuals (unpublished data). This result suggests there is polymorphism for the presence of a paralogous copy within *S. vulgaris*, although heteroplasmy involving a rare haplotype is also plausible.

The acceleration in *atp9 *appears to be common to most of *Silene*/*Lychnis*. In contrast, most of the other *Sileneae *genera exhibit more conventional substitution rates for *atp9*, although their rates are still elevated on average. This pattern is consistent with an *atp9*-specific increase in substitution rate very early in the divergence of *Silene*, which may have been magnified by further accelerations in local areas of the genus.

A previous study of mitochondrial substitution rate variation across the seed plant phylogeny identified a handful of individual species exhibiting elevated divergence in one gene but not others [4]. Our observations of rate variation in *atp9 *within *Silene *indicate that such gene-specific effects can be maintained across large clades of species over millions of years. We also found that these effects can occur quite locally. Most notably, *S. nutans *exhibited an *R*_*S *_value of 80 SSB for *atp1 *(a rate that exceeds all other species for that gene), but it showed no sign of acceleration in *nad9 *or *cox3 *(Figure 3). A number of other species showed more modest rate increases in *atp1 *and/or *cox3 *without correlated accelerations in other genes. These patterns may reflect local mutational effects within the genome. Alternatively, given the mounting evidence for recombination in plant mtDNA [41,46,54] and the existence of rate variation both within and among species [7], rate discrepancies between genes may be the result of recombination between genomes with different mutational histories. Finally, the possibility of nuclear transfer for a gene such as *atp1 *in *S. nutans *should also be considered [4].

### Evolution at synonymous and non-synonymous sites

Despite the massive variation in synonymous substitution rates among genes and species, we found that rates of non-synonymous substitution generally remained low (although there was a positive correlation between *R*_*N *_and *R*_*S *_across branches: Figure 3, Table 3). Across genes, *R*_*S *_values vary by 9 to 41-fold (depending on whether the six species with apparent genome-wide accelerations are included), while *R*_*N *_values vary by only 2 to 3-fold (Table 2). As a result, there is an apparent negative relationship between *R*_*S *_and the ratio of non-synonymous to synonymous changes (ω). While *R*_*S *_is commonly interpreted as a measure of the mutation rate, *ω *is used as an estimate of the intensity/efficacy of purifying selection (*i.e*. "the selective sieve" [55]). Under these interpretations, our data would suggest that genes experiencing high mutations rates also face greater purifying selection. In contrast, the opposite pattern has been observed in comparisons of nuclear genes in mammals [56].

The relationship between *R*_*S *_and *ω *among mitochondrial genes in *Silene *should be confirmed in a larger sample, because we have examined only 4 loci in the present study, and *atp9 *may generally be subject to strong purifying selection [57]. Whether *R*_*S *_and *ω *can be reliably interpreted as measures of mutation rate and purifying selection depends on the distribution of fitness effects for mutations at synonymous and non-synonymous sites, which are not well understood in plant mitochondrial genomes. These distributions will dictate how the synonymous and non-synonymous substitution rates scale with the mutation rate.

In a comparison of sequence divergence in 15 protein-coding mitochondrial genes between angiosperms and the liverwort *Marchantia*, Laroche *et al*. [57] found much greater variation among genes in *d*_*N *_than in *d*_*S*_--the opposite of what we observed. This discrepancy highlights the importance of phylogenetic scale in these studies. Across deep nodes in the land plant phylogeny, local differences in gene-specific mutation rates are apparently averaged out, and variation in the magnitude of purifying/positive selection among genes becomes the primary determinant of evolutionary rates. In contrast, at the local phylogenetic scale of our study, the signature of gene-specific differences in mutation rate is apparently maintained.

Because of the high variance and abundance of 0 values associated with short branches in our analysis, it is difficult to test for the same relationship between *R*_*S *_and *ω *across lineages that we observed across genes. We did see, however, that removing the rapidly evolving branches from each tree raised the *ω *ratio for all four genes, suggesting that the same pattern may hold. Mower *et al*. [4] conducted a broad phylogenetic survey of seed plants in which short branch lengths would be expected to be less of a problem. Their data showed a strong negative relationship between *R*_*S *_and *ω *across lineages (see also [53]). Therefore, it appears that major increases in mitochondrial synonymous substitution rate--either gene or taxon-specific--are accompanied by a less than proportional increase in non-synonymous substitution rate such that the effects of an apparent increased mutational pressure on amino acid sequences are greatly dampened.

### Uncertainty in divergence time estimates

We used molecular clock based methods to estimate divergence times in our *matK *gene tree. The estimated ages were generally older than estimates from two previous studies [4,30]. The discrepancy between these studies is likely attributable to two major differences. First, there is simple difference in calibration age between our study and the analysis of Mower *et al*. (2007), which utilized an age of 38 Myr for the *Beta*/*Silene *divergence derived from a broader molecular clock analysis of the angiosperms [58]. This date appears to be in conflict with our fossil calibration point, as all three of our analyses estimate the age of the *Beta*/*Silene *split to be at least 52 Myr old. This distinction, however, cannot explain the contrasting results between our study and that of Frajman *et al*. [30], because we used essentially the same calibration point. Instead, we note that there was a significant difference in sampling schemes between these two studies. Our focus on the genus *Silene *produced a very imbalanced topology with much denser branching in certain parts of the tree than others. In contrast, Frajman *et al*. [30] utilized a much more balanced phylogenetic sampling. Because it is easier to detect multiple substitutions at the same site in regions with lots of branching, there is a tendency to estimate longer branch lengths in species-rich parts of a phylogeny (the "node density effect" [59]). This effect may contribute to our older age estimates within the tribe *Sileneae*. Because of this potential bias, the uncertainty over calibration points and the many assumptions associated with molecular clock based dating, it is important to stress that the divergence times used in this analysis should be considered only as approximations.

Divergence time estimates are necessary to calculate absolute substitution rates, so dating uncertainty should be considered in comparing absolute rates across studies. For example, re-calibrating node ages within *Silene *to correspond with the 12 Myr divergence time for the genus estimated by Frajman *et al*. [30] would increase our substitution rate estimates by approximately 50%. In relative terms, however, our three dating analyses were quite consistent within *Sileneae*. Therefore, our estimates of proportional variation in substitution rate across species and genes are less sensitive to dating method.

## Conclusion

Based on our analysis of mitochondrial divergence within the tribe *Sileneae*, we conclude that mutational acceleration is not restricted to a single species nor is it completely confined to a small number of high rate lineages. The patterns of divergence in *atp9 *illustrated that elevated rates have been maintained throughout much of the genus *Silene *for at least one mitochondrial gene, highlighting a complex gene × species interaction in the distribution of rate variation. The diversity in phylogenetic and genomics scale suggests that there is no simple rule or single mechanism underlying mutation rate variation in plant mitochondrial genomes. Elucidating the mechanistic forces that shape mutation rate variation should represent a high priority in the field of plant mitochondrial genomics. *Silene *was targeted for this in-depth sampling of species-level mitochondrial divergence because of *a priori *knowledge of the rate acceleration in *S. noctiflora*. Determining whether the patterns of rate variation among species and among genes in *Silene *are broadly representative of angiosperm genera or represent something unique about the molecular evolution of *Silene *will require similar levels of sampling in taxa that currently show no evidence of rate increase.

## Methods

### Study species

*Silene *(Caryophyllaceae) comprises approximately 700 predominantly herbaceous species that vary substantially in life history and breeding system [60]. The genus has become a model system for diverse areas of research with a particular focus on the molecular evolution of organelle genomes, including studies of population genetics [61,62], organelle transmission [25,27], evolutionary rates [4,7,35,45], and cytoplasmic male sterility [26,44]. *Silene *belongs to the tribe *Sileneae*, which has been the subject of extensive and ongoing phylogenetic analysis [30-32,63-65]. Taxa were selected so as to represent major groups that will appear in a forthcoming revised taxonomy of the genus (Oxelman *et al*. in prep). For this study, we used a combination of field collected samples and preserved herbarium specimens along with previously published sequence data. Sample collection and voucher information are summarized in Table 1.

### DNA extraction, PCR and Sequencing

We extracted total genomic DNA from each sample. For silica-dried samples and herbarium specimens, we followed the protocol described by Oxelman *et al*. [31] and performed subsequent purification using the Qiagen QIAquick Purification Kit protocol, Ultra Silica Bead kit (ABgene), or GFX PCR DNA and Gel Band Purification Kit (Amersham Biosciences). For fresh tissue samples, extractions were performed using the Qiagen Plant DNeasy Kit.

We PCR amplified the full-length coding sequence of the chloroplast gene *maturase K *(*matK*) and portions of four mitochondrial protein coding genes: *ATP synthase subunit 1 *(*atp1*), *ATP synthase subunit 9 *(*atp9*), *cytochrome c oxidase subunit 3 *(*cox3*) and *NADH dehydrogenase subunit 9 *(*nad9*). [See Additional file 8 for PCR primer sequences.]

PCR products were cleaned with Exonuclease I and shrimp alkaline phosphatase (USB Corporation), cycle sequenced with BigDye v3.1 (Applied Biosystems), and analyzed on an ABI 3130 × l capillary sequencer. Automated basecalls were edited manually using published *Beta vulgaris *sequences as a reference for reading frame, and sequences were assembled into contigs using Sequencher v4.5 (Gene Codes). All sequences obtained for *S. sorensenis *were identical to those from *S. involucrata*, so *S. sorensenis *was excluded to simplify subsequent analysis. DNA sequences have been submitted to GenBank [see Additional file 9 for accession numbers]. Sequence alignments were generated using the Clustal function imbedded in MEGA v4.0 [66] and edited manually [see Additional file 10].

### *matK *phylogenetic analysis and dating

We estimated the phylogeny of our sample based on the *matK *dataset, using both maximum likelihood (ML) and maximum parsimony (MP) criteria in PAUP* v4.0b10 [67]. In addition to the species listed in Table 1, our analysis also included *matK *sequences from GenBank for the following outgroups: *Beta vulgaris *(Amaranthaceae), *Illecebrum verticillatum *(Caryophyllaceae) and *Scleranthus perennis *(Caryophyllaceae). Our ML search employed a GTR+Γ substitution model with fixed parameter values identified based on an analysis of our full *matK *dataset (including outgroups) using the AIC method in ModelTest v3.7 [68]. The ML topology was identified with a heuristic search using the TBR branch swapping algorithm, the MULTREES option in effect, and random addition of sequences with 10 replicates. A MP bootstrap analysis based on 1000 replicate datasets was performed using the same heuristic search settings except with MULTREES off. We performed ML and MP analyses in the same fashion for each mitochondrial gene.

We used three different techniques to estimate divergence times from our *matK *gene tree: (1) a Bayesian relaxed clock model implemented in BEAST v1.4.8 [69], (2) a penalized likelihood (PL) method, and (3) the Langley-Fitch (LF) method. The latter two methods were both performed in r8s v1.71 [70]. The LF model is a maximum likelihood strict molecular clock method that enforces a constant substitution rate over the entire tree. The other two methods allow for rate variation among branches. The PL approach assumes that rates are correlated across adjacent branches and penalizes models that require rapid rate changes within the tree. In contrast, the BEAST analysis constrained the rate variation among branches to a lognormal distribution but placed no restriction on correlations between adjacent branches. All dating analyses incorporated an extra outgroup, *Nepenthes glabrata *(Nepenthaceae), which was added solely to determine the position of the root along the *Beta vulgaris *branch. It was pruned from the resulting trees and discarded from all subsequent analyses. We used a calibration time of 34 million years (Myr) for the split between *Scleranthus *and *Sileneae*, which corresponds to the recent analysis of Frajman *et al*. [30] and the fossil evidence described therein.

The BEAST analysis was conducted with a GTR+Γ model of substitution with 4 rate categories, empirical base frequencies and a birth-death process tree prior. We defined a monophyletic ingroup to include all species except *Nepenthes*, *Beta *and *Illecebrum*. The calibration date was effectively fixed by specifying a normal distribution with mean of 34 and standard deviation of 0.00001 as the prior for the time to most recent common ancestor (TMRCA) of the pre-defined ingroup. We ran 3 MCMC chains of length 50 million each with trees saved every 25,000 iterations. The first 1000 trees (50%) from each chain were discarded as burn in, and chains were combined after verifying convergence among runs. We generated a maximum credibility tree with mean node heights as well as a tree that was constrained to the 70% bootstrap consensus topology from our parsimony analysis. BEAST reported the estimated node ages along with the associated 95% high probability densities (HPDs), as well as the posterior probability for each node.

For the PL and LF analyses in r8s, we used the 70% parsimony bootstrap consensus topology with branch lengths optimized under ML in PAUP*. We fixed the root age of the tree to an arbitrary value of 1 and calibrated the resulting output tree with the 34 Myr fossil age for the *Scleranthus*/*Sileneae *split. Both analyses utilized the TN search algorithm with 10 restarts, 10 time guesses, and the checkgradient option on. The cross-validation procedure was used to determine an optimal smoothing parameter of 0.0022 for the PL analysis. Error in divergence times for both methods was estimated based on the distribution of 100 bootstrap replicate datasets, following the recommendations in the r8s documentation.

### Estimating *d*_*N *_and *d*_*S*_

We estimated the branch lengths of *matK *and all 4 mitochondrial genes individually in terms of synonymous (*d*_*S*_) and non-synonymous (*d*_*N*_) substitutions per site, using a codon-based model of substitution within the codeml application in PAML v4.0 [71]. We also analyzed a concatenation of all 4 mitochondrial genes and a concatenation of *atp1*, *cox3 *and *nad9 *only. Tree topologies were constrained based on the *matK *70% parsimony bootstrap consensus. Codon frequencies were determined by an F1x4 model. The parameters values for *ω *and transition/transversion ratio were estimated from the data with initial values of 0.4 and 2 respectively. Separate *ω *values were estimated for each branch. As in the dating analysis, *Nepenthes glabrata *was used as an outgroup in the *matK *dataset to identify the position of the root along the *Beta vulgaris *branch. *Arabidopsis thaliana *served a similar purpose for the mitochondrial genes. These outgroups were pruned from the resulting trees and not considered further. Because the process of C-to-U RNA editing can bias the estimation of *d*_*N *_and *d*_*S *_values in plant mitochondrial genomes [72], we excluded all codons known to undergo RNA editing in *Beta vulgaris *[73].

In the *matK *dataset, 6 species (*Heliosperma pusillum*, *S. conica*, *S. paradoxa*, *S. samojedora*, *S. seoulensis *and *S. conoidea*) produced sequences with an apparent frameshift indel in one of two homopolymer regions, raising the possibility that we sequenced pseudogenes in these species. In addition, *S. otites *did not have a start codon at the conserved position in the *matK *alignment. These sequences showed little indication of elevated rates or other abnormal substitution patterns, and their phylogenetic placement was consistent with *a priori *information. Therefore, they were retained in the dataset, and codons that contained frameshift indels were removed to keep all sequences in frame.

For the mitochondrial dataset, we obtained sequences for *atp1*, *cox3*, and *nad9 *from all sampled species, but only a subset of *atp9 *sequences were successfully generated. For 5 of the 74 species in our sample, we failed to successfully amplify and sequence *atp9*, and an additional 9 species appeared to have multiple *atp9 *copies. In the latter group, sequencing electropherograms indicated the presence of two different nucleotides in between 1.2 and 19.5% of sites. These samples were excluded from the analysis.

### Estimates of absolute substitution rates (*R*_*N *_and *R*_*S*_)

Following the basic methodology described by Cho *et al*. [5], absolute substitution rates (substitutions per site per year) can be obtained by dividing branch lengths (defined in terms of substitution per site) by the age of the branch. We calculated branch ages using the divergence times estimated by BEAST. We divided the *d*_*N *_and *d*_*S *_values reported by PAML for each branch by the respective branch age to obtain absolute substitution rates in terms of non-synonymous and synonymous sites (*R*_*N *_and *R*_*S*_, respectively). Standard errors for *R*_*N *_and *R*_*S *_were calculated as described by Parkinson *et al*. [6] where the standard errors for node ages were approximated as one quarter of the 95% HPD. Pearson correlations coefficient for *R*_*N *_and *R*_*S *_values across and within genes were calculated with PROC CORR in SAS Software v9.1.

## Authors' contributions

DBS conceived of the study, participated in its design, conducted the bulk of the sequencing and data analysis, and drafted the manuscript. BO participated in design of the study, collected and identified specimens, and helped with data analysis and drafting of the manuscript. AR collected and identified specimens and helped with sequencing, data analysis, and drafting of the manuscript. DRT participated in design of the study and helped with data analysis and drafting the manuscript. All authors read and approved the final manuscript.

## Supplementary Material

Additional file 1 **Estimated divergence times (in millions of years) from three different dating methods**. [See Additional file 2 for definitions of node names]. Click here for file

Additional file 2 **Names for internal nodes**. The labels to the right of each node correspond to the names used in Additional files 1, 3, and 4. Click here for file

Additional file 3 **Detailed data on *d*_*N*_, *d*_*S*_, *R*_*N*_, *R*_*S *_and associated error for each gene and concatenated dataset (all species)**. Each row corresponds to a phylogenetic branch defined by its basal node and derived node/tip. *R*_*N *_and *R*_*S *_values are in terms of SSB. Approximated standard errors (SEs) are provided for *R*_*N *_and *R*_*S*_. Note that SE approximations are undefined and reported as 0 for any absolute rate estimate of 0. This table does not include *atp9 *because it was not sequenced in all species. [See Additional file 2 for definitions of node names]. Click here for file

Additional file 4 **Detailed data on *d*_*N*_, *d*_*S*_, *R*_*N*_, *R*_*S *_and associated error for each gene and concatenated dataset (*atp9 *subset)**. Same setup as Additional file 2. Only the 61 species for which *atp9 *was sequenced are included, so that data for *atp9 *and the concatenation of all four mitochondrial genes could be presented. [See Additional file 2 for definitions of node names]. Click here for file

Additional file 5 **Maximum likelihood trees for each of the 4 mitochondrial genes (generated without topological constraint)**. Parsimony bootstrap values are noted to the left of the corresponding node. Only values > 0.5 are shown. Branch lengths are in terms of substitutions per site. Click here for file

Additional file 6 **Maximum likelihood tree for *matK *dataset**. Branch lengths are in terms of substitutions per site. Click here for file

Additional file 7 **BEAST analysis of *matK *dataset with unconstrained topology**. Time scale is in millions of years. Posterior support is shown to the right of each node. Click here for file

Additional file 8 **Sequences and references for PCR primers**. Nucleotide sequences (5' to 3') of primers used for PCR amplification and DNA sequencing. Click here for file

Additional file 9 **GenBank accession numbers for all sequences**. Accession numbers in bold are not from the voucher listed in Table 1. Click here for file

Additional file 10 **Table S3**. Alignments for each gene in FASTA format. Click here for file
