# Phylogenetic analysis of mitochondrial substitution rate variation in the angiosperm tribe *Sileneae*

**DOI:** 10.1186/1471-2148-10-12

**Published:** 2010-01-14

**Authors:** Daniel B Sloan, Bengt Oxelman, Anja Rautenberg, Douglas R Taylor

**Affiliations:** 1 Department of Biology, University of Virginia, Charlottesville, VA, USA; 2 Department of Plant and Environmental Sciences, University of Gothenburg, Sweden; 3 Department of Systematic Biology, EBC, Uppsala University, Sweden

## Correction

The published version of the article entitled "Phylogenetic analysis of mitochondrial substitution rate variation in the angiosperm tribe *Sileneae*" [1] contained an error in the legend of Figure 2. The third sentence of the legend should have read, "Values to the right of each node show parsimony bootstrap support and Bayesian posterior probability (in that order) for the corresponding clade." The authors regret the error and apologize for any confusion it may have caused.
