# Supplementary figures and images for: Phylogenetic analysis of mitochondrial substitution rate variation in the angiosperm tribe Sileneae
Source: BMC Evol Biol. 2009 Oct 31;9:260. doi: 10.1186/1471-2148-9-260 (PMC2777880; doi:10.1186/1471-2148-9-260)

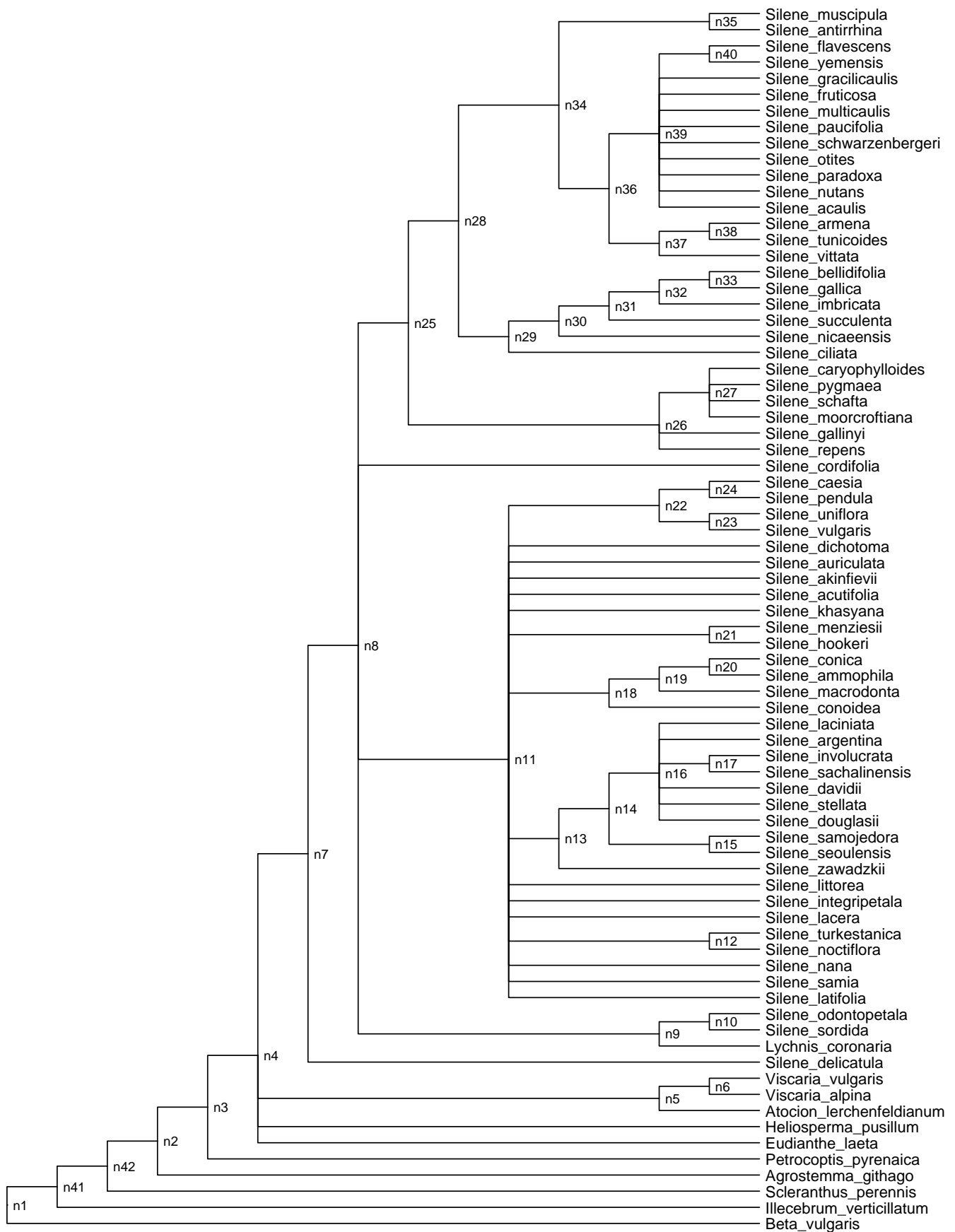

Supplement: Additional file 2 — Names for internal nodes. The labels to the right of each node correspond to the names used in Additional files 1, 3, and 4. [file 1471-2148-9-260-S2.pdf]

*nad9*

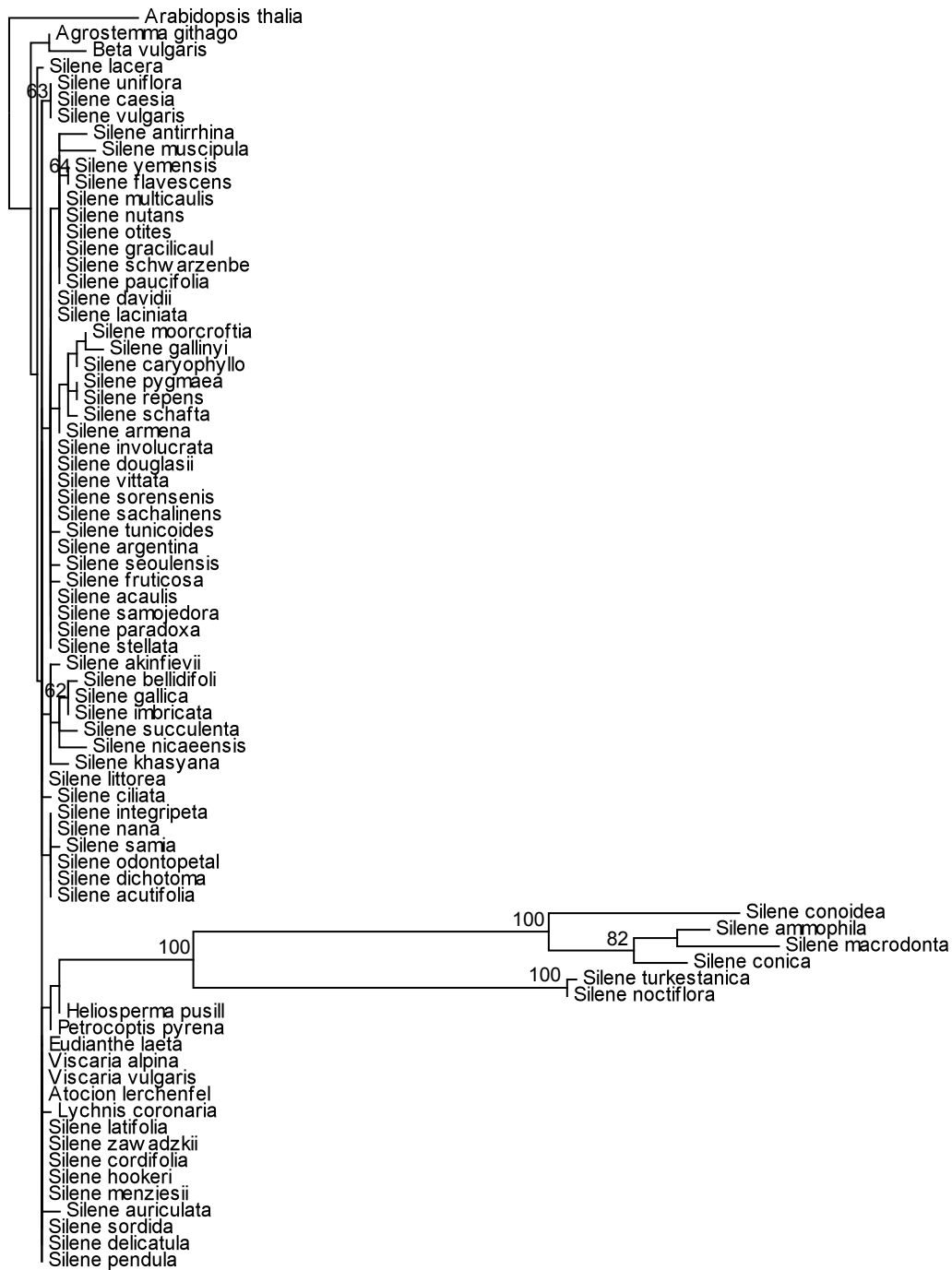

cox3

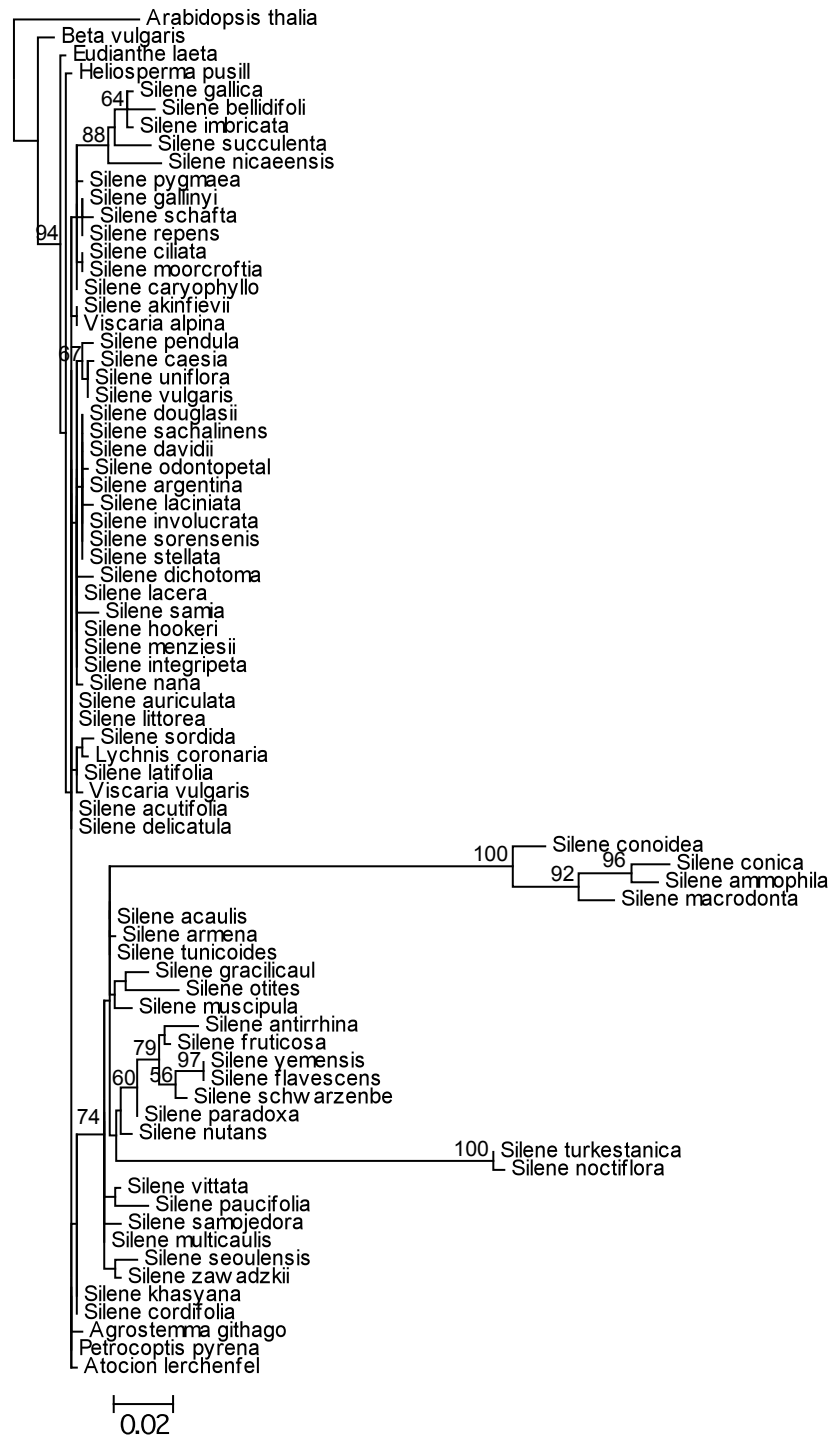

*atp1*

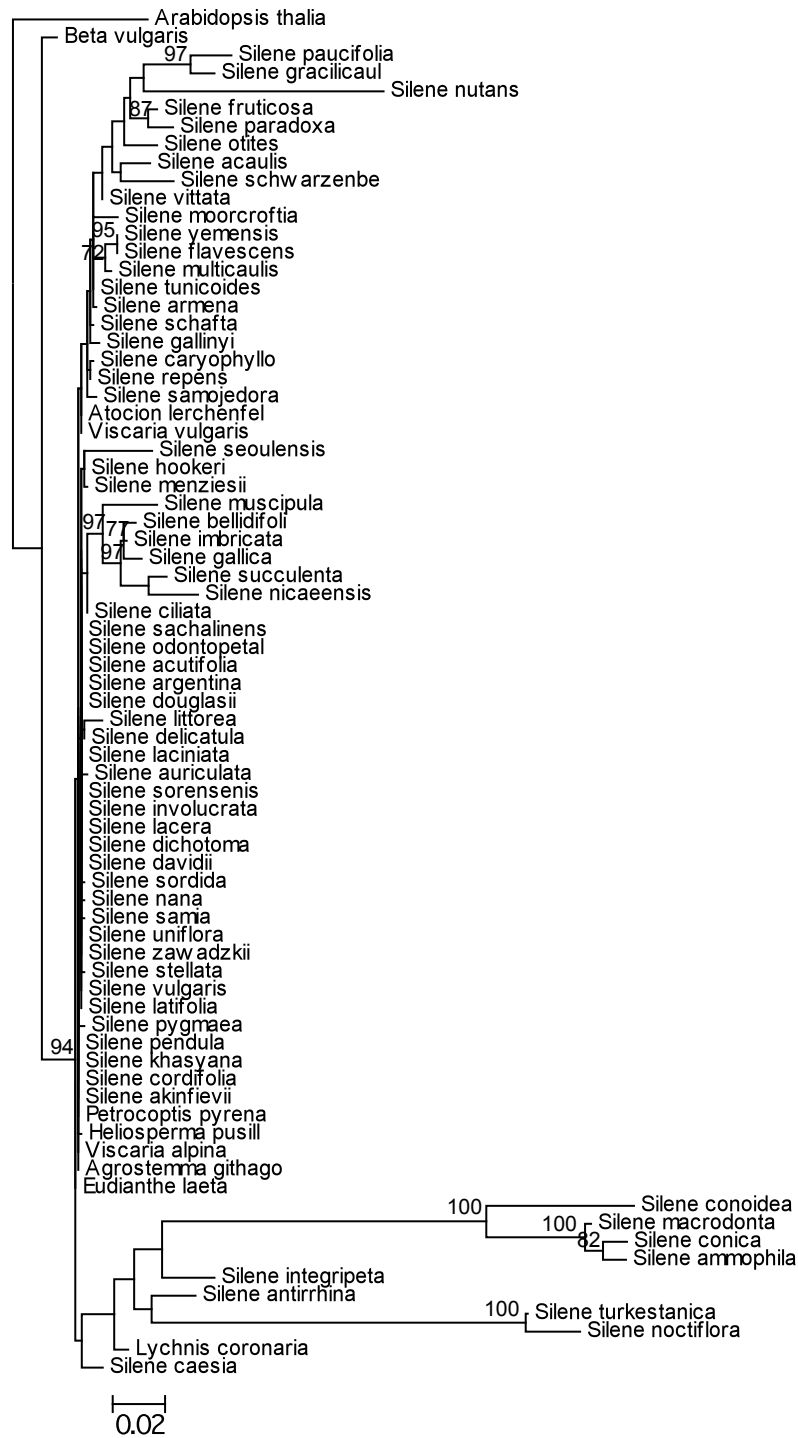

*atp9*

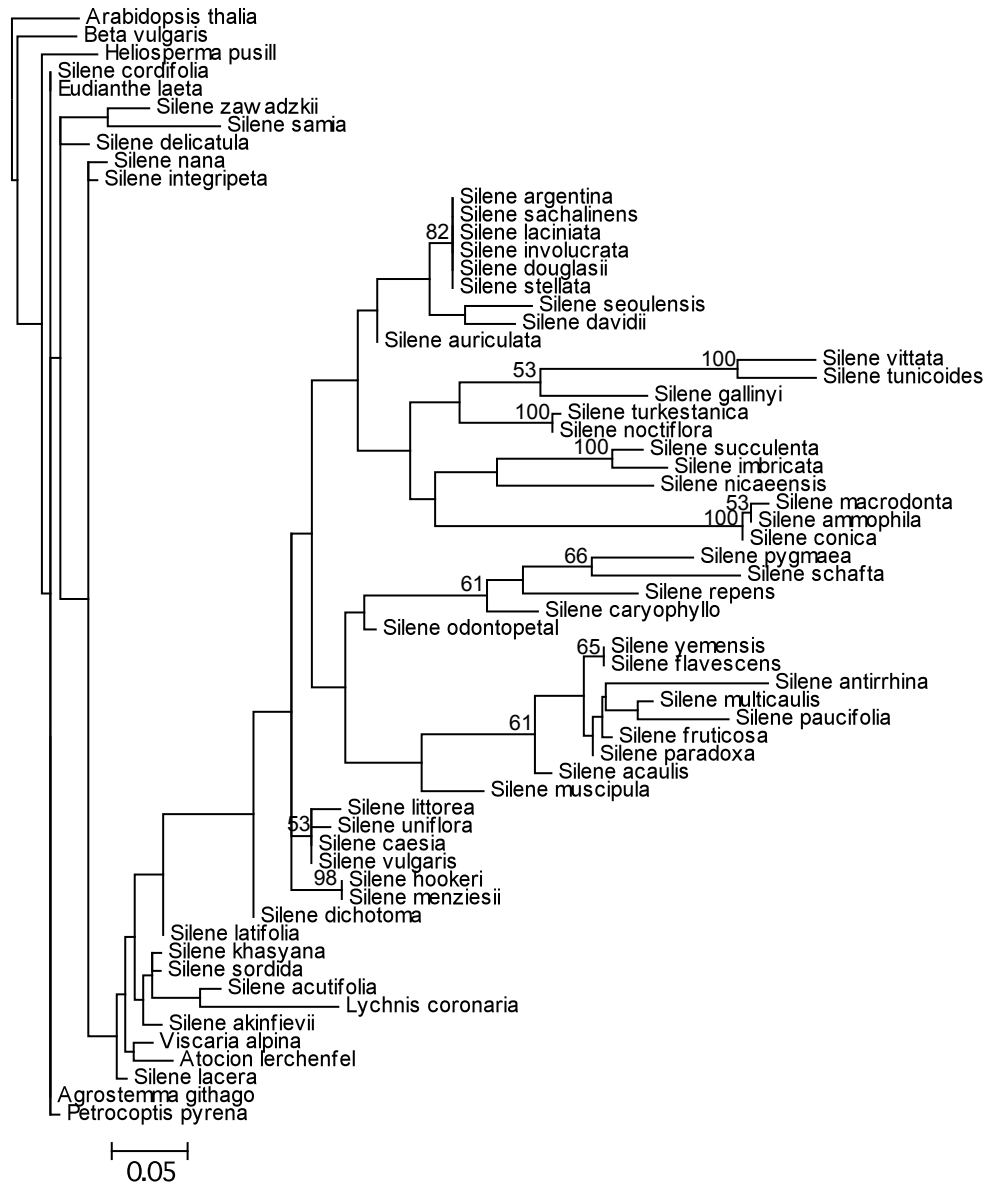

Supplement: Additional file 5 — Maximum likelihood trees for each of the 4 mitochondrial genes (generated without topological constraint). Parsimony bootstrap values are noted to the left of the corresponding node. Only values > 0.5 are shown. Branch lengths are in terms of substitutions per site. [file 1471-2148-9-260-S5.pdf]

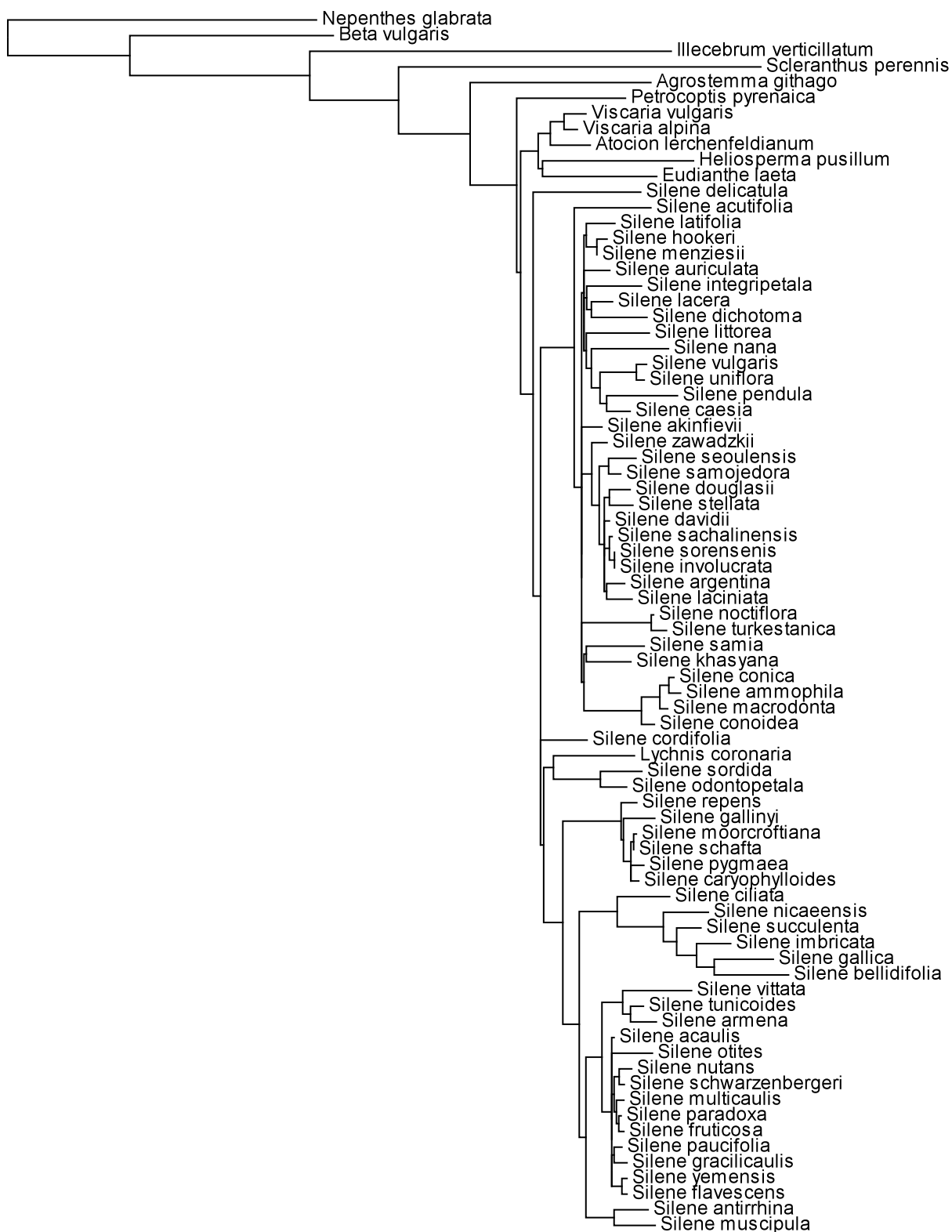

Supplement: Additional file 6 — Maximum likelihood tree for matK dataset. Branch lengths are in terms of substitutions per site. [file 1471-2148-9-260-S6.pdf]

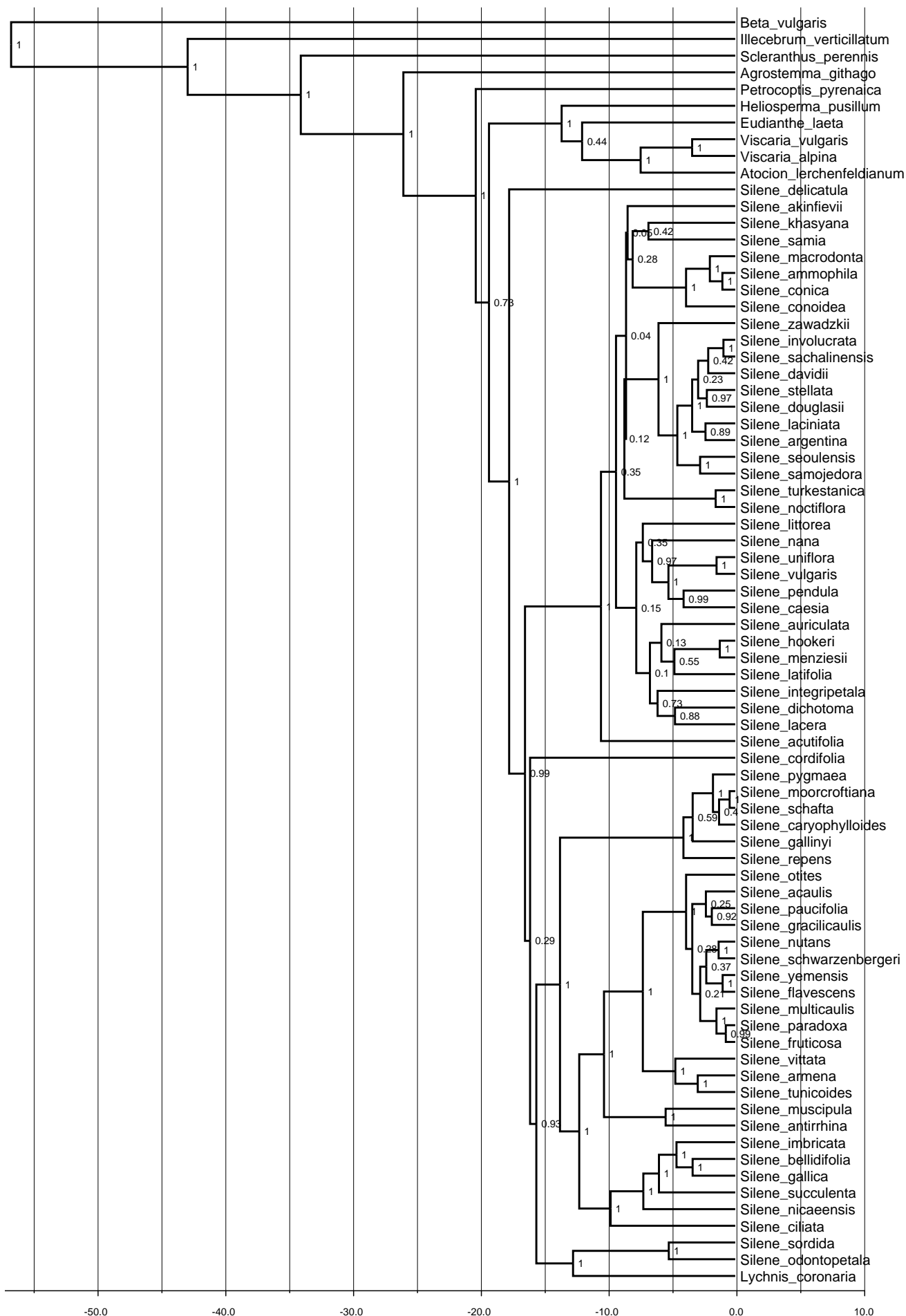

Supplement: Additional file 7 — BEAST analysis of matK dataset with unconstrained topology. Time scale is in millions of years. Posterior support is shown to the right of each node. [file 1471-2148-9-260-S7.pdf]
